# Oxidative Stress and Neural Dysfunction in Gastrointestinal Diseases: Can Stem Cells Offer a Solution?

**DOI:** 10.1093/stcltm/szad063

**Published:** 2023-09-29

**Authors:** Rhian Stavely, Leah C Ott, Lauren Sahakian, Niloufar Rashidi, Samy Sakkal, Kulmira Nurgali

**Affiliations:** Department of Pediatric Surgery, Pediatric Surgery Research Laboratories, Massachusetts General Hospital, Harvard Medical School, Boston, USA; Department of Pediatric Surgery, Pediatric Surgery Research Laboratories, Massachusetts General Hospital, Harvard Medical School, Boston, USA; Institute for Health and Sport, Victoria University, Melbourne, Australia; Institute for Health and Sport, Victoria University, Melbourne, Australia; Institute for Health and Sport, Victoria University, Melbourne, Australia; Institute for Health and Sport, Victoria University, Melbourne, Australia; Department of Medicine Western Health, The University of Melbourne, Melbourne, Australia; Regenerative Medicine and Stem Cell Program, Australian Institute for Musculoskeletal Science (AIMSS), Melbourne, Australia

**Keywords:** oxidative stress, reactive oxygen species, gastrointestinal, mesenchymal stem cell, enteric nervous system, multipotent stromal cell

## Abstract

Oxidative stress is involved in many gastrointestinal (GI) disorders as either the primary pathogenesis (radiation, chemotherapy, toxicity, ischemia-reperfusion) or a secondary driving force of disease progression (inflammation and diabetes). The GI tract is innervated intrinsically by the enteric nervous system (ENS) with a diverse role in maintaining gut homeostasis and GI motility. Complications in the physiological functioning of the ENS results in GI dysfunction that can result in debilitating sequelae from dysmotility greatly impacting quality of life and leading to potentially fatal complications. Therapeutics to remedy either oxidative stress or enteric neuronal dysfunction are severely limited, resulting in a critical gap in clinical care for GI disease and neurointestinal complications. Stem cell therapies have shown great promise in the treatment of several gut disorders via mechanisms including cell regeneration, anti-inflammatory activity, providing trophic support, and emerging evidence of antioxidant and neuroprotective functions. The potential of mesenchymal stem cell (MSC) therapies and recent evidence of their antioxidant and neuroprotective activity in several GI conditions are discussed. Finally, future therapeutic aspects of stem cell-based tools for combatting oxidative stress and enteric neuropathies in GI disease are considered.

## Graphical Abstract

**Graphical Abstract:**
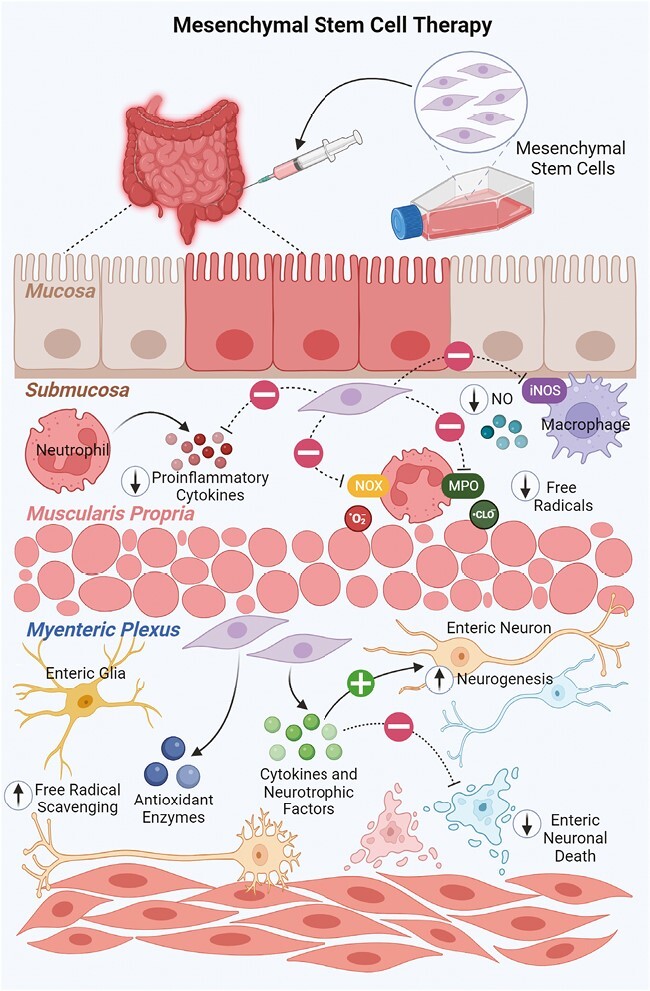

**Significance Statement**

This review discusses the emerging link between oxidative stress and enteric nervous system dysfunction, highlighting current knowledge around mechanisms of enteric neuropathies implicated in gastrointestinal disorders. It also discusses existing research on the multiple antioxidant mechanisms of mesenchymal stem cell therapies in gut disorders. The opportunity of utilizing the antioxidant properties of stem cells to ameliorate neural dysfunction in gastrointestinal diseases and future therapeutic aspects of stem cell-based therapies for combatting oxidative stress in gastrointestinal diseases and associated neuropathy are considered.

## Introduction

Oxidative stress is critical in the pathophysiology of various gastrointestinal (GI) disorders arising from chronic infections, inflammation, malignancies, diabetes, ischemia-reperfusion injury, GI toxicities due to chronic alcohol consumption, radiotherapy, nonsteroidal anti-inflammatory, and chemotherapeutic drugs. Many GI conditions also exhibit damage to the enteric nervous system (ENS), including altered structure, neuroinflammation, neuronal hyperexcitability/altered signaling properties, and enteric neuropathy, which can lead to additional GI complications. With limited treatment options to target these pathophysiological mechanisms, the potential of stem cell therapies for these conditions is explored.

## Oxidative Stress

After the great oxygenation event 2.4 billion years ago, cellular organisms took advantage of the unique reactivity of O_2_ to produce massive amounts of energy that could sustain the complex multicellular lifeforms observed today. While these oxidizing properties are advantageous for cellular metabolism and oxidative phosphorylation, the same reactivity is also detrimental to cellular structures and countermeasures via antioxidant defense mechanism became critical to existence.^1,2^ Due to the dual beneficial and harmful roles of oxygen and its subsequent products of reactive oxygen species (ROS), the balance of the reduction-oxidation (redox) environment has become essential to regulate many physiological and pathophysiological mechanisms in cellular biology.^3,4^ Although ROS and similar reactive products such as reactive nitrogen species (RNS) are often considered damaging compounds, they have also been incorporated into normal physiological cellular functions and, at moderate levels, have important roles in mediating apoptosis and cellular senescence, act as secondary messengers in intracellular signaling cascades, modify transcriptional processes, have a role in oxygen sensing and regulate smooth muscle tone.^2^ ROS also serve a protective function when wielded by various immune cells, purposefully generated by enzymatic reactions to confer their bactericidal properties.^5-7^ This is particularly pertinent in the GI tract which houses around 100 trillion bacteria in humans. Nitric oxide (NO) is enzymatically formed by 3 nitric oxide synthase (NOS) isoforms and has been incorporated into several physiological functions. Particularly in the gut, enteric neurons produce NO as an important neurotransmitter to evoke smooth muscle relaxation allowing successful propulsion of luminal contents along the digestive system.^8^ Although ROS and RNS are required for normal physiological functions, disturbances in this delicate balance to a highly pro-oxidative environment can result in oxidative stress.

Oxidative stress refers to a deviation from the physiological redox state and an increase in pro-oxidants, or free radicals, that structurally change lipids, proteins, and DNA in a way that causes pathology or damage to a cell.^9^ The subsequent cell/tissue damage and activation of apoptotic cell signaling cascades can then lead to inflammation and chronic disease.^10^ Oxidative stress results from an imbalance in either the generation or sequestering of ROS and RNS. There are various pathways of ROS formation, notably mitochondrial reduced nicotinamide adenine dinucleotide phosphate (NADPH) oxidase (NOX) and xanthine oxidase (XOD) are considered to be the major cellular sources of the superoxide (O_2_^−^) radical anion.^11^ Other major ROS and RNS with important biological functions include the hydroxyl radical (•OH), hydrogen peroxide (H_2_O_2_), hypochlorous acid (HOCl), nitric oxide (NO), and peroxynitrite (ONOO−).^4^ The immune system heavily relies on these ROS/RNS to elicit a normal immune response. These compounds can be generated directly from immune cells through enzymes including NOX, inducible nitric oxide synthase (iNOS), and myeloperoxidase (MPO) or indirectly as metabolic byproducts of other inflammation-associated enzymes such as lipoxygenase (LOX) and cyclooxygenase (COX).^5,12,13^ However, during sustained inflammation, these compounds can lead to cell death and disturbed cellular functions. Excess ROS production specifically by the mitochondria can also have important pathological consequences. O_2_^−^ generated from complexes of the electron transport chain (ETC) are highly reactive and can damage the mitochondrion.^14^ Excess O_2_^−^ and NO can react to form the ONOO^−^ compound, which can extenuate the destructive properties of ROS to proteins, lipids, and DNA.^15^ The detoxification of O_2_^−^ into H_2_O_2_ is mediated by superoxide dismutase (SOD).^13^ However, H_2_O_2_ can also be generated in various metabolic processes and by dual oxidases (DUOX)_._^16^ Both H_2_O_2_ and O_2_^−^ can diffuse across cell membranes and affect many cellular processes.^1,17^ While H_2_O_2_ is more stable than O_2_^−^, its detoxification is crucial, as it possesses a weak peroxide bond that makes it susceptible to reacting with metals, such as Fe^2+^, to generate reactive •OH through the Fenton reaction.^18^ Catalase (CAT) is a major contributor to the detoxification of H_2_O_2_ and a final step in antioxidant enzyme reactions converting H_2_O_2_ to innocuous water and O_2_. However, the glutathione system is also important to the detoxification of diverse oxidative species via its scavenging properties and enzymatic reactions. Glutathione peroxidase catalyzes the detoxification of ONOO^−^ and H_2_O_2_ by reduced glutathione (GSH) and subsequently produces glutathione disulfide (GSSG).^19^ Glutathione reductase (GSR) reduces GSSG back to monomeric GSH, creating a cycle that buffers against excessive accumulation of ROS. In the gut, heme oxygenase-1 (HO-1) may also play an important antioxidant role, such as through the catabolism of heme, which contributes to the damage mediated by oxidative stress, and the production of carbon monoxide (CO), ferritin and bilirubin which themselves possess antioxidant properties.^20-23^

Oxidative stress is implicated in several acquired or congenital GI disorders, as well as adverse effects of various therapeutics and procedures on the GI tract and can lead to the progression of chronic conditions and the development of debilitating GI sequelae that contribute to severe pathology. While many free radicals and pathways implicated in oxidative stress converge in GI disorders, each condition has unique mechanisms leading to oxidative stress which warrants consideration to develop effective therapies and apply them in a diligent manner. While an in depth review on these mechanisms is outside the scope of this article, Bhattacharyya, et al^24^ and Vona et al^25^ offer great insight into how oxidative stress is implicated in GI disorders affecting both the mucosa and the muscular layers.

## Antioxidant Effects of Mesenchymal Stem Cells in the Gut

**Figure 1.**
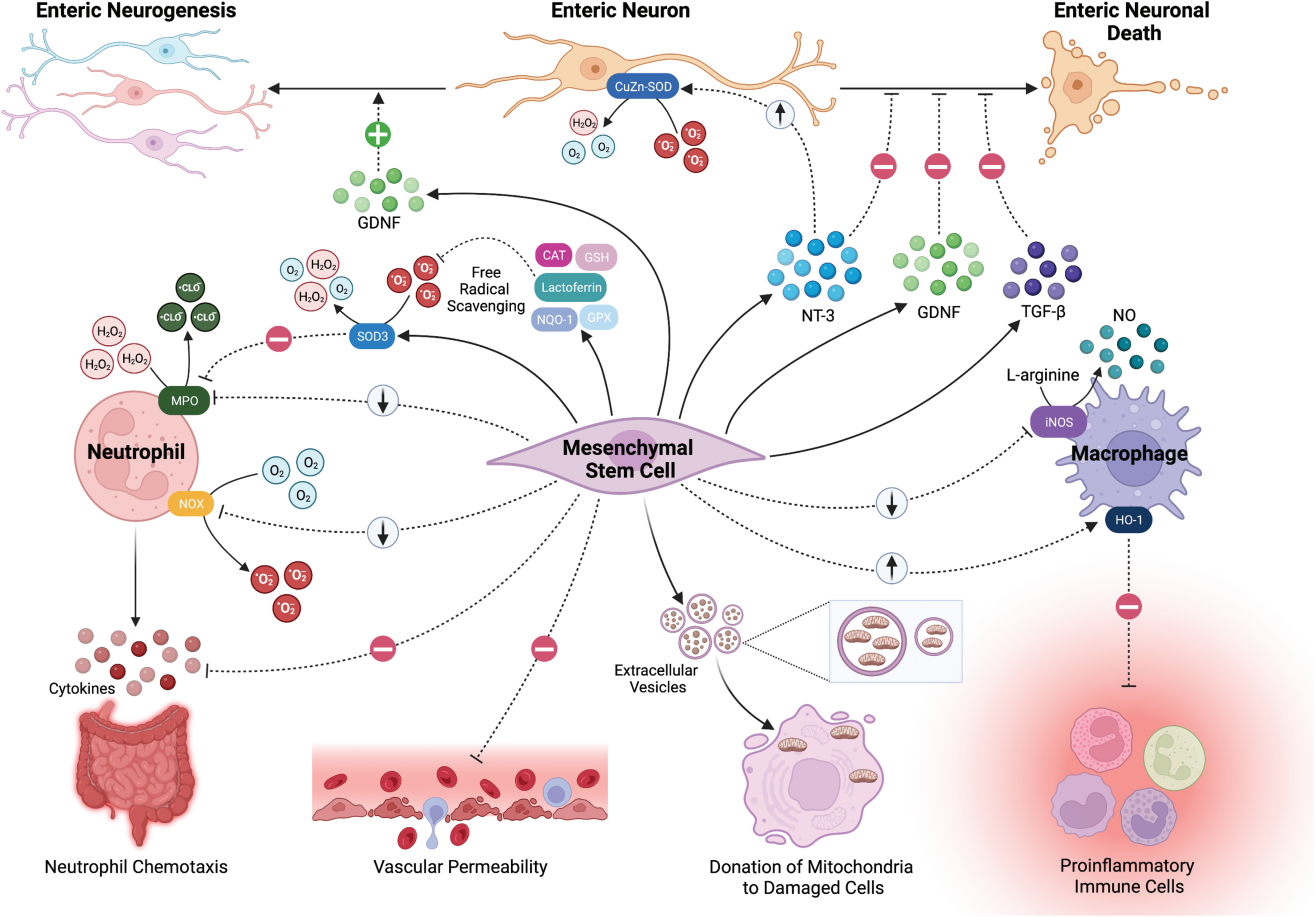
Summary of antioxidant, anti-inflammatory, and neuroprotective properties of mesenchymal stem cells (MSCs) which may alleviate gastrointestinal oxidative stress. MSCs have been shown to limit damage from neutrophils in experimental colitis by suppressing their recruitment to the colon, as well as inhibiting expression of myeloperoxidase (MPO) and activation of the respiratory burst via superoxide dismutase (SOD)3. MSCs can also suppress iNOS and NOX-1/2 expression by proinflammatory immune cells, such as activated macrophages, and induce expression of HO-1, attenuating intestinal inflammation. They are known to secrete and stimulate the activity of various antioxidants, including lactoferrin, catalase (CAT), glutathione (GSH), glutathione peroxidase (GPx), and NADPH dehydrogenase quinone 1 (NQO-1), enhancing free radical scavenging. In addition, MSCs have been shown to limit tissue damage and vascular permeability following IRI and can donate mitochondria to damaged cells via extracellular vesicles. Beyond their anti-inflammatory and antioxidant properties, MSCs have been shown to secrete neurotrophic factors, such as neurotrophin-3 (NT-3) and glial-derived neurotrophic factor (GDNF), to directly protect neurons from oxidative stress. These neurotrophic factors then promote upregulation of endogenous antioxidant defenses in neurons, such as CuZn-SOD. In addition, MSCs produce transforming growth factor-β (TGF-β), which has been shown to exert neuroprotective effects on the ENS. Finally, MSCs have been shown to stimulate enteric neurogenesis, likely through the secretion of neurotrophic factors, without transdifferentiating into enteric neurons themselves. Created with BioRender.com.

While stem cells are often associated with their regenerative capabilities, they also possess other unique characteristics that may be leveraged therapeutically, such as their trophic secretome, anti-inflammatory properties, and ability to reduce oxidative stress. The most well-studied sources of stem cells are mesenchymal stem cells (multipotent stromal cells, MSCs) isolated from bone marrow (BM), adipose tissue (AT), and the umbilical cord. MSCs exhibit potent antioxidant properties.^26^ These antioxidant effects are multifaceted and can include direct scavenging of ROS, induction of endogenous antioxidant defenses in host tissues, immunosuppression limiting ROS production, prevention of the dysfunction in mitochondrial bioenergetics, and donation of mitochondria to damaged cells.^26^ The multiple antioxidant mechanisms of stem cell therapies and the prospect for transplanted cells to target specific organs and supply local antioxidants for prolonged periods of time might offer a significant advantage over systemic small-molecule antioxidants. MSCs react to oxidative stress and in response upregulate their antioxidant defenses.^26^ This raises the possibility of MSCs acting as implantable local sensors to the redox status that correct oxidative stress upon elevated stimuli but do not interfere with the physiological messenger functions of ROS during periods of homeostasis. Evidence for the antioxidant effects and utilization of stem cells for the treatment of gut diseases has accumulated with studies applying MSCs in models of experimental colitis, intestinal ischemia and colorectal carcinogenesis, where they reduce oxidative stress by inhibiting lipid peroxidation and increasing antioxidant capacity^27-30^ (Table 1). The antioxidant mechanisms of MSCs in the intestine could be attributed to the direct scavenging of ROS by MSCs, promoting endogenous antioxidant defenses and modifying activation states, or preventing the recruitment of ROS-generating immune cells (Fig. 1).

**Table 1.** Effects of MSCs on oxidative stress and enteric neuropathy in the GI tract.

Model	Methodology	Effect of MSCs	Citations
Colorectal cancer	Intrarectal administration of BM-MSCs in a rat model of 1,2-dimethylhydrazine (DMH)-induced colorectal carcinogenesis	Oxidative stress ↓ Epithelial dysplasia ↓ Lipid peroxidation ↓ iNOS ↑ CAT activity	Alkhuriji et al^30^
Epithelial barrier permeability	Caco-2 cells stimulated by H_2_O_2_ and treated with MSC conditioned media	Oxidative stress ↓ Permeability with MSC in a cell impedance assay - Effects occurred in paracrine manner	Barry et al^31^
Ischemia-reperfusion injury	72 hours post intravenous and intrajejunal administration of AT-MSCs	Oxidative stress ↓ Oxidized proteins ↓ ROS generating enzymes; MPO, iNOS, NOX-1, and NOX-2 ↑ Antioxidant enzymes; NQO-1, GSR, HO-1	Chang et al.^32^ Inan et al.^27^
	7 days post intravenous and submucosal injection of BM-MSCs	↓ Lipid peroxidation after 1 day ↓ SOD, glutathione peroxidase, CAT - More favorable effects by local administration	
Inflammatory bowel disease	IL-10 knockout mouse model of chronic colitis with intravenous administration of BM-MSCs	Oxidative stress ↓ Lipid peroxidation ↓ O_2_^.−^, H_2_O_2_	Jung et al.^33^ Sun et al.^28^ da Costa Gonçalves et al.^34^ Anderson et al.^35^ Sala et al.^36^ Banerjee et al.^37^ Robinson et al.^38^ Stavely et al.^39^ Stavely et al.^40^ Robinson et al.^41^
	DSS mouse model with intravenous injection of BM-MSC	↓ Lipid peroxidation ↑ SOD	
	DSS model in mice with intravenous injection of AT-MSC	↑ SOD activity ↑ Glutathione	
	DSS model in mice with intravenous injection of AT-MSC	↓ iNOS in macrophages	
	DSS model in mice with intraperitoneal injection of BM-MSC	↑HO-1	
	DSS model in immunodeficient mice with intravenous injection of UC-MSCs	↓ MPO ↓ Endoplasmic stress Enteric Neuropathy	
	TNBS model of colitis in guinea pigs with intraluminal application of BM-MSCs, AT-MSCs, or conditioned media.	↑ Enteric neurons - Prevented neuronal subpopulation shifts - Paracrine manner	
Enteric neuropathy	Benzalkonium chloride (BAC)-induced aganglionosis followed by local pyloric injection of BM-MSCs into the muscularis propria	Enteric Neuropathy ↑ Gastric emptying ↑ Enteric neurogenesis from endogenous progenitors - MSCs did not transdifferentiate into enteric neurons	Lin et al.^42^ Fan et al.^43^
Diabetic gastroparesis	Intraperitoneal injection of placenta-derived MSC into non-obese diabetic mice	Enteric Neuropathy ↑ nNOS neurons ↑ ICCs	Park et al.^44^

Abbreviations: AT-MSC: adipose tissue-derived mesenchymal stem cell; BAC: benzalkonium chloride; BM-MSC: bone marrow-derived mesenchymal stem cell; CAT: catalase; GSR: glutathione reductase; H_2_O_2_: hydrogen peroxide; HO-1: heme oxygenase-1; ICC: interstitial cells of Cajal; IL-1:, interleukin-10; iNOS: inducible nitric oxide synthase; MPO: myeloperoxidase; nNOS: neuronal nitric oxide synthase; NOX: NADPH-oxidase; NQO-1: NADPH dehydrogenase quinone 1; O_2_^−^: superoxide; ROS: reactive oxygen species; SOD: superoxide dismutase; UC-MSC: umbilical cord-derived mesenchymal stem cell.

Considering the bidirectional codriving effects between oxidative stress and the immune response, the amelioration of oxidative stress by MSCs in intestinal inflammation could be explained by either an anti-oxidative or anti-inflammatory mechanism; however, there is evidence for concurrency in these events. For example, neutrophil infiltration is a prominent feature in experimental colitis, and these cells produce a respiratory burst of free radicals including O_2_^−^, H_2_O_2_, and hypochlorite generated by NOX and MPO, leading to oxidative stress-induced damage.^45,46^ Many studies have demonstrated that MSCs reduce the levels of MPO in experimental colitis as a surrogate measurement of neutrophil recruitment. Therefore, MSCs could prevent oxidative stress from neutrophils via suppression of inflammatory pathways upstream of neutrophil chemotaxis to the intestine. Conversely, MSCs have been shown to directly suppress respiratory burst and MPO expression in neutrophils via the scavenging properties of MSC-derived SOD3.^47^ Similarly, it has been reported that MSC treatments can reduce the expression of iNOS and its product NO, which can react with ROS to form compounds that cause cellular damage via nitration.^29,48^ In intestinal inflammation, ‘activated’ macrophages consistent with an M1-like phenotype are the major producers of iNOS. MSCs are capable of modifying the polarization state of macrophages to phenotypes with reduced expression of iNOS,^35^ which may then play a downstream role in preventing tissue damage by reactive species produced during the immune response. In fact, MSCs injected in the intraperitoneal cavity have been shown to aggregate with immune cells and promote high levels of HO-1.^36^ This suggests that MSCs can not only prevent the pro-oxidative properties of the immune system but also modify leukocytes to promote defenses against oxidative stress-induced tissue damage. While the attenuation of colitis by MSCs has been previously linked to modification of T-cell responses, studies in immunodeficient mice with dextran sodium sulfate (DSS)-induced colitis linked the therapeutic properties of MSCs with reduced MPO levels and further demonstrated a reduction in endoplasmic stress and the activation of the unfolded protein response, which is likely to contribute to oxidative stress via the generation of ROS in intestinal inflammation.^37,49^

In further support of the ability of MSCs to promote defenses against ROS generation in intestinal inflammation, levels of the antioxidant glutathione are increased after MSC treatments,^34^ likewise, MSCs increased the level or activity of the SOD, which scavenges the highly reactive O_2_^−^.^28,34^ In the interleukin-10 (IL-10) knockout model of chronic colitis, BM-MSCs were also found to decrease the reactive species O_2_^−^, H_2_O_2_, and suppress subsequent lipid peroxidation.^33^ Although the relationship between endogenous MSC cellular sources and IBD is unclear, MSCs isolated from the AT of CD patients exhibit high levels of lactoferrin, which has ROS scavenging properties.^50^ The conditioned medium had an enhanced therapeutic capacity in experimental colitis compared to MSCs from uninflamed controls with low-lactoferrin levels. Moreover, the administration of lactoferrin reproduced many of the therapeutic effects of the MSC-conditioned medium.^50^ Therefore, MSCs themselves could be activated by the inflammatory environment to promote their antioxidant tissue-protective effects in IBD.

The development of colorectal cancer is a critical complication of IBD with a 60% increase in incidence compared to the general population.^51^ This elevated risk of colorectal cancer has been attributed to the oxidative stress response, which induces DNA damage and excessive mutations leading to the formation of cancerous cells.^52^ This is an important consideration for MSC therapies, as the trophic and anti-inflammatory properties can have a protumorigenic effect on established colorectal cancers in the gut,^53^ albeit reports are mixed and may be dependent on tumor heterogeneity.^54^ Regardless, MSC treatments have been demonstrated to reduce the risk of developing the initial neoplasm and tumorigenesis in the context of inflammation-induced colorectal cancer, which suggests that MSC treatments can have a beneficial effect if administered prior to colorectal cancer formation.^55,56^ Notably, MSCs have also demonstrated the capability to reduce colon carcinogenesis in models of chemically induced mutagenesis, which was associated with their antioxidant properties. In a model of 1,2-dimethylhydrazine (DMH)-induced colorectal carcinogenesis, rectal application of BM-MSCs prevented epithelial dysplasia with a concurrent decline in lipid peroxidation, iNOS expression and an increase in CAT activity.^30^ Considering oxidative stress is instrumental in driving mutations responsible for carcinogenesis, future studies should determine whether the antioxidant properties of MSCs can also contribute to suppressing the development or progression of neoplasms in intestinal inflammation.

Several studies have also reported favorable outcomes utilizing MSC therapies for intestinal ischemia-reperfusion injury with oxidative stress considered to play a primary role in the pathophysiology of this condition. In one study, the effects of autologous AT-MSCs were investigated in a rat model of mesenteric artery occlusion.^32^ Mesenteric arteries were clamped for 30 minutes followed by both intravenous and intrajejunal administration of MSCs. After 72 hours, AT-MSCs reduced levels of oxidized proteins and the expression of immune system ROS-generating enzymes, including MPO, iNOS, NOX-1, and NOX-2. Furthermore, MSC treatments resulted in the upregulation of several antioxidants, including NADPH dehydrogenase quinone 1 (NQO-1), GSR, glutathione peroxidase, and HO-1. These results suggest that MSCs can act by 2 mechanisms in this condition: (1) suppression of ROS generated by the immune system and (2) upregulation of antioxidant defenses. In a similar study of small bowel ischemia-reperfusion injury in the rat, the superior mesenteric artery was clamped for 45 minutes, and reperfusion injury was assessed over a 7-day time course.^27^ BM-MSCs were administered either systemically or by local injection to the intestinal submucosa immediately after the ischemia-reperfusion procedure. In this study, MSC treatments were shown to reduce oxidative stress, as demonstrated by lipid peroxidation, within 1 day. In addition, more favorable effects were observed in the local MSC-treated group. Levels of antioxidant enzymes SOD, glutathione peroxidase, and CAT were elevated in rats with ischemia-reperfusion injury, which is representative of the physiological oxidative stress response. However, unlike the study by Chang et al,^32^ levels of antioxidants were reduced by MSC treatments in the Inan et al^27^ study rather than further increased. Therefore, the antioxidant mechanisms remain unclear; however, these results could be explained by the rapid prevention of oxidative stress and, thus, a diminished oxidative stress response.

MSCs were investigated in a model of hemorrhagic shock and trauma, which involves elements of tissue ischemia driving epithelial and vascular permeability.^31^ In this model, MSCs ameliorated the histopathology of the small intestine and vascular permeability within hours, further demonstrating their ability to act rapidly as therapeutic agents. The potential mechanisms were further investigated in vitro in a model of epithelial barrier permeability using Caco-2 cells stimulated by H_2_O_2_. In this system, the MSC-conditioned media reversed the permeability of the epithelium, indicating that MSCs could act via an antioxidant mechanism or at least alleviate oxidative stress-associated damage; furthermore, these results suggest that the MSC secretome alone can harbor these properties.^31^

## Mesenchymal Stem Cells for the Treatment of Enteric Neuropathy

The gastrointestinal (GI) tract is innervated intrinsically by the enteric nervous system (ENS), a division of the autonomic nervous system (ANS), which influences both the severity and progression of GI dysfunction in inflammation and chemotherapy, thereby emerging as a potential, novel therapeutic target for GI disorders.^57,58^ The ENS consists of 2 plexuses, containing both neurons and glial cells; an outer myenteric plexus and an inner submucosal plexus.^59^ The myenteric plexus consists of ganglia between the outer longitudinal and inner circular smooth muscle layers of the bowel, controlling their contraction and relaxation; in addition, it interacts with tissue-resident muscularis macrophages.^60^ Meanwhile, the submucosal plexus is nestled between the muscle and epithelium and serves to regulate mucosal functions, immune cell migration, and blood flow.^60,61^ Thus, any damage to the ENS has severe consequences for these physiologic GI functions.

Several GI disorders exhibit dysfunction in the ENS including altered structure, neuroinflammation, neuronal hyperexcitability/altered signaling properties, and enteric neuropathy or aganglionosis, which consequentially leads to an imbalance in gut homeostasis and usually dysmotility or aperistalsis. These include idiopathic gastroparesis, Hirschsprung’s disease, esophageal achalasia, Chagas disease, pyloric stenosis, MNGIE, and subtypes of chronic intestinal pseudo-obstruction, which are collectively referred to as neurointestinal disease.^62^ Other GI conditions that exhibit similar abnormalities in neutrally mediated functions secondary to another insult, such as IBD, diabetic gastroparesis, and chemotherapy-induced GI side effects, also exhibit a neurointestinal disease component associated with significant morbidity. Either can result in debilitating sequelae from dysmotility that need to be rectified to improve quality of life and prevent potentially fatal complications such as impaction or perforation. Currently, there is a major gap in therapeutic agents designed to alleviate neural dysfunction in GI disorders that could be fulfilled by MSCs as shown in models-associated with enteric neuropathies (Table 1).

In a model of gastroparesis in non-obese diabetic mice, intraperitoneal injection of placenta-derived MSCs prevented the loss of nNOS neurons in gastric tissues.^44^ Notably, in several GI conditions there is thought to be a preferential loss of nNOS neurons in enteric neuropathy due to their susceptibility to oxidative injury,^63^ as the nitrosative product NO can react to form the compound peroxynitrite which damages proteins, lipids, and DNA.^15^ MSCs have demonstrated clear neuroprotective effects via antioxidant mechanisms in peripheral and central nervous system neuropathies, including oxaliplatin-induced peripheral neuropathy in the dorsal root ganglia and sciatic nerve as well as ischemia, inflammation, or chronic alcohol toxicity in the brain.^64-67^ Therefore, it may be plausible to utilize MSCs for enteric neural dysfunction in conditions associated with oxidative stress. In studies by our group, BM- and AT-MSCs from allogeneic or xenogeneic sources applied rectally in the trinitrobenzene sulfonic acid (TNBS)-induced colitis model were found to reduce exaggerated expression of nNOS-expressing enteric neurons and prevent inflammation-induced neuropathy.^39-41^ These therapeutic effects were similarly exerted when utilizing only the MSC-conditioned media, indicating that these effects were mediated by paracrine factors.^38^ In these studies, the therapeutic effects of MSCs are likely attributed to their ability to suppress the initial causes of neuropathy or to modify the neuronal responses to these stimuli. For example, MSCs have been shown to reduce oxidative stress in these models, which appears to be an important mediator of enteric neuropathy in inflammatory conditions.^68^ However, MSCs are also potent producers of neurotrophic factors which can directly protect neurons from oxidative stress. Neurotrophin-3 (NT-3) and glial-derived neurotrophic factor (GDNF) have previously been shown to prevent the death of enteric neurons in models of oxidative stress utilizing H_2_O_2_ and menadione.^69,70^ These mechanisms may include promoting endogenous antioxidant defences within neurons, as observed by enhanced CuZn-SOD in enteric neurons in response to GDNF.^71^ In addition, MSCs secrete the cytokine transforming growth factor-β1 (TGF- β1), which attenuated the loss of myenteric neurons in vitro consistent with its known neuroprotective effects in disorders of the CNS.^40,72,73^ Given that MSCs have been shown to prevent peripheral neuropathies induced by chemotherapeutic agents via antioxidant mechanisms,^74,75^ the application of these cells to alleviate chemotherapy-induced enteric neural dysfunction and GI side effects, such as nausea and dysmotility, warrants future exploration.

The studies described above provide a case for utilizing MSCs as a cell therapy to prevent enteric neuropathy; however, an important consideration remains regarding how enteric neurons can be replenished after enteric neuropathies have developed. In a model of benzalkonium chloride-induced enteric neuropathy in the pylorus, MSCs have demonstrated favorable effects.^42^ In this study, BM-MSCs were injected into the muscularis propria of the pylorus 3 days after the initial ENS ablation. MSC treatments restored gastric emptying and promoted de novo regeneration of enteric neurons in the pylorus, without trans-differentiation of the transplanted BM-MSCs into neurons. These effects were suggested to be a result of neurotrophic factors, such as GDNF, which was elevated in tissues treated with MSCs.^42^ Further work by the authors clarified that BM-MSC treatments promoted regeneration of the enteric nervous system by the endogenous enteric neural progenitor cells.^43^ Other strategies to replace enteric neurons after damage to the ENS could include the use of enteric neural stem cells (ENSCs), subcutaneous adipose tissue neural stem cells (SAT-NSCs), and induced pluripotent stem cells (iPSCs), which have shown to be effective at reestablishing the ENS in models of colonic aganglionosis (Hirschsprung’s disease), gastroparesis, and necrotizing enterocolitis.^76-79^

## Future of Antioxidant and Neuroprotective Stem Cell Therapeutics in the Gut

Oxidative stress and neural dysfunction are important features of GI disease and so far, proven difficult to translate into effective treatments. Currently, MSCs are the most extensively studied stem cell therapeutics for GI disease and have demonstrated potent antioxidant and neuroprotective properties in the gut. Although MSCs are the most widely studied cellular therapeutics, high expression of antioxidant enzymes and neurotrophic factors can also be observed in the transcriptome of ENSCs, enteric mesenchymal cells, and SAT-NSCs.^76,80^ The potential utility of neural-crest cells to alleviate GI pathology via mechanisms other than transdifferentiation into enteric neurons is unexplored, but potentially feasible if they act in a similar manner to the endogenous glia to protect enteric neurons. Neural crest-derived stem cells appear to have excellent engraftment rates in the intestine, which could provide an advantage over MSC therapies.^76,80^

There are promising data for the utilization of MSCs as targeted therapeutics to alleviate oxidative stress in the intestine and ENS; however, several opportunities for improvement are being explored to enhance their therapeutic effects or make treatments more clinically feasible. Due to the heterogeneous nature of autologous stem cells, well-characterized allogeneic cell lines of MSCs are being explored for therapeutic application.^81^ Notably, stem cell-based products could be used as a substitution for live MSCs as both the conditioned media and secreted exosomes/vesicles from these cells have been shown to reduce oxidative stress. Extracellular vesicles in particular can house similar antioxidant enzymes as MSCs, such as peroxiredoxin 1-6, SOD1-2, CAT, and thioredoxin.^82^ Many studies have also demonstrated that MSCs can be used as vehicles for the delivery of bioactive cytokines and enzymes via genetic engineering, which could be leveraged for the treatment of neuropathy in GI diseases. For example, MSCs genetically modified to express excessive levels of the antioxidant enzyme HO-1 have enhanced effects on intestinal ischemia-reperfusion injury.^83^ Likewise, overexpression of SOD2 in MSCs was found to improve their ability to rescue brain tissue from neuroinflammation in traumatic brain injury.^84^

Exploration into the use of MSCs to treat either oxidative stress or enteric neural dysfunction has been primarily limited to colitis, ischemia-reperfusion injury, and gastroparesis. Several GI diseases could benefit from the antioxidant properties of MSC therapies. The initial data in experimental models looks promising in regards to the utility of stem-cell therapies to treat GI conditions associated with oxidative stress and neural dysfunction. However, the practical implementation of stem cell therapies requires careful deliberation as pathophysiology and clinical needs for each condition vary considerably. For example, for acute injuries such as postoperative ileus, NEC, and bowel resection with primary anastomosis, it may be unnecessary and even confer unacceptable risk to have cells implanted in the gut long-term. These conditions may benefit from impermanent treatments such as conditioned media, exosomes, or even allogeneic/xenogeneic MSCs that are eventually rejected by the host, reducing the risk of off-target effects. Another important consideration is the timing of treatment for specific conditions. IBD flares may be treated acutely, however, due to the chronic course of the disease and high risk of relapse, chronic maintenance therapies may be necessary; therefore transplantable cells could be promising in this space. Other conditions in which cell therapies might be protective when used preventatively could include those at high risk of developing oxidative stress-associated cancers such as inflammation-induced colorectal cancer and Barrett’s esophagus. The success of radiotherapy is dependent on oxidative stress and interfering with this mechanism to limit side effects could lead to severe consequences. In this instance, cell therapies may offer benefits when applied after the initial treatment in the recovery phases. The use of MSCs in conditions associated with impaired neuromuscular function, such as hypertrophic pyloric stenosis and Triple A syndrome esophageal achalasia, also warrants attention, considering promising results for MSC-mediated local repair of the ENS in vivo. However, it is unknown whether other cell types, such as enteric neural stem cells, would be better suited for such clinical applications. The ability of MSCs to restore mitochondrial bioenergetics or donate mitochondria themselves could also have potential implications for the treatment of mitochondrial-related GI neuropathies, such as MNGIE.

## Conclusions

Oxidative stress and nervous system injury both prove challenging to address therapeutically. Stem-cell therapies could offer promise for these difficult-to-treat conditions. MSCs in particular have demonstrated efficacy in suppressing oxidative stress in models of IBD, ischemia-reperfusion injury, and the development of CRC. Evidence of enteric neuroprotection or neurotrophic regeneration of the ENS after MSC treatment is observed in models of IBD, diabetic gastroparesis, and pyloric stenosis caused by ENS ablation. Other sources of stem cells, such as enteric neural stem cells and subcutaneous adipose tissue-derived neural stem cells, may also warrant exploration for their neuroprotective properties. Finally, stem cell-based therapies such as the stem cell secretome and exosomes could provide a novel solution to wielding the therapeutic effects of stem cells without the risks associated with cell implantation in conditions that only require acute intervention.

## Data Availability

The data underlying this article will be shared on reasonable request to the corresponding author.
